# Cortical Thickness and Hippocampal Volume in Vascular and Non-vascular Depressed Patients

**DOI:** 10.3389/fpsyt.2021.697489

**Published:** 2021-07-14

**Authors:** Dakota A. Egglefield, Sophie Schiff, Jeffrey N. Motter, Alice Grinberg, Bret R. Rutherford, Joel R. Sneed

**Affiliations:** ^1^The Graduate Center, City University of New York, New York, NY, United States; ^2^Queens College, City University of New York, Queens, NY, United States; ^3^Department of Psychiatry, Columbia University College of Physicians and Surgeons, New York, NY, United States; ^4^Division of Geriatric Psychiatry, New York State Psychiatric Institute, New York, NY, United States; ^5^Department of Neurology, Albert Einstein College of Medicine, Bronx, NY, United States

**Keywords:** vascular depression, white matter hyperintensities, cortical thickness, hippocampal volume, mild cognitive impairment

## Abstract

**Background:** Reduced cortical thickness and hippocampal volume are prevalent markers of late life depression as well as mild cognitive impairment (MCI) but are conspicuously absent in the vascular depression (VD) literature. The present study aimed to determine differences in cortical thickness and hippocampal volume between VD and non-VD patients.

**Methods:** Participants were enrolled in an 8-week open treatment antidepressant trial. Forty-one depressed individuals aged 50 and older underwent brain magnetic resonance imaging at baseline and were classified as VD or non-VD. Cortical thickness values for the left and right entorhinal, parahippocampal, and precuneal cortices, as well as left and right hippocampal volume, were linearly regressed on VD status to determine mean differences between VD and non-VD. Covariates included site, age, sex, and mean thickness or intracranial volume.

**Results:** No statistical differences were found between VD and non-VD patients in cortical thickness of the bilateral precuneal, entorhinal, or parahippocampal cortices, or hippocampal volume (*p* > 0.001).

**Conclusions:** The absence of statistical differences in gray matter between VD and non-VD patients raises several diagnostic, etiological, and developmental possibilities, namely that VD may not be connected with other late-life psychiatric illnesses such as MCI or dementia and that vascular disease may not be a common etiological risk factor for depression and dementia. Larger datasets, prospective longitudinal studies, and cognitively intact controls are needed to further address these types of questions.

## Introduction

Depression in late life is often associated with cognitive impairment and has been shown to increase risk for developing dementia (1), but the heterogeneity of late life depression (LLD) complicates a complete understanding of this relationship (2). One strong possibility is that depression is a causal risk factor or else moderator of neurodegenerative processes leading to Mild Cognitive Impairment (MCI) and dementia. Consistent with this possibility is that while some depressed patients remain cognitively normal, others experience neuropathology associated with Alzheimer's Disease (AD), and later convert to MCI or AD (2). On the other hand, it is possible that the apparent depression-dementia causal relationship is confounded by vascular disease, as vascular disease is a risk factor for and common comorbidity in both disorders (3). Vascular depression (VD) has traditionally been proposed as a subtype of LLD (4) defined by cerebrovascular disease (5, 6) (manifested by the presence of deep white matter hyperintensities (DWMHs) on MRI) and characterized by executive dysfunction (5, 7–9). The VD hypothesis proposes that vascular risk factors lead to DWMHs which disconnect prefrontal cortical regions from subcortical regions, resulting in the onset of depressive symptoms, executive dysfunction, and poor response to antidepressant treatment (6, 10).

There is significant phenomenological and neuropathologic overlap between the vascular subtype of LLD and MCI. Clinically, an individual presenting with characteristic symptoms of vascular depression could also be conceptualized as being diagnosed with a subtype of MCI with depression, particularly non-amnestic single-domain MCI (11). Reduced cortical thickness and hippocampal volume are salient features of MCI and AD that often predate symptom onset (12–15). Specifically, the medial temporal lobe (12) and precuneus gyrus (14, 16) are among the first areas to deteriorate in cognitively normal individuals who later convert to MCI or dementia. Gray matter atrophy in similar brain regions is also apparent in LLD: depressed older adults compared to healthy controls show bilateral cortical atrophy in the frontal, parietal, and temporal lobes, as well as lower hippocampal volumes (17, 18). Poor antidepressant treatment response in geriatric depressed patients is associated with smaller hippocampal volumes (19) and poor response to psychotherapy is associated with thinner bilateral parahippocampal and left precuneal cortices, among other regions (20).

Of note, comparing LLD patients to healthy controls leaves open the possibility that findings of reduced cortical thickness and hippocampal volume observed in LLD patients may be driven by the component of the sample with vascular subtype of LLD. To help clarify whether volumetric brain changes associated with MCI/dementia are more related to the presence of a mood disorder or rather the presence of significant vascular disease, a sample of patients with the vascular subtype of LLD would need to be compared to depressed individuals without vascular risk. If reduced cortical thickness in medial temporal brain regions and hippocampal atrophy were to be observed among individuals with vascular depression compared to depressed patients without vascular lesions, this would suggest a common causal model of the depression-dementia relationship in which vascular disease leads to both conditions.

As studies of cortical thickness and hippocampal volume have been conspicuously absent in VD research, a comparison of these features between VD and non-vascular depressed patients could further elucidate the VD construct as well. Toward this end, evaluating structural brain differences between these two groups of late life depressed patients across different definitions of vascular depression could be very informative. Thus, the goal of this study was to evaluate the presence of gray matter atrophy in VD compared to non-vascular LLD (non-VD). In keeping with the idea that vascular disease may play a critical etiologic role in both VD and MCI, the present study hypothesized that VD patients will show decreased cortical thickness in the entorhinal and parahippocampal (medial temporal lobe regions) and precuneal cortices, and lower hippocampal volumes, compared to non-VD patients.

## Materials and Methods

### Participants

Patients from two sites, New York State Psychiatric Institute and Harlem Hospital Center, participated in an 8-week open antidepressant medication trial (escitalopram or duloxetine) for older adults with depression. Patients underwent a comprehensive neuropsychological evaluation at baseline and week 8 as well as received baseline imaging. The full study protocol has been described elsewhere (9). Patients were eligible if they were 50 years or older; had a current diagnosis of major depressive disorder, dysthymia, or depression not otherwise specified; and had a Hamilton Rating Scale for Depression (HRSD) score of ≥14 at their initial screening, corresponding to at least a moderate level of depressive severity. Patients were excluded from participating in the study if they had other Axis I diagnoses such as bipolar disorder, obsessive compulsive disorder, psychotic disorder, or current substance abuse or dependence within the past year; were actively suicidal or had a past suicide attempt within the last 6 months; or had a Mini Mental Status Exam (MMSE) score lower than 24.

### MRI and Cortical Thickness

Patients underwent structural MRI at baseline. Images were acquired on a GE Signa 3 Tesla whole body scanner with the following sequences: (1) 3-Plane localizer repetition time (TR) = 23.4 ms, echo time (TE) = 1.7 ms, flip angle = 30°, bandwidth = 31.3 MHz, field of view (FOV) = 24 × 24 cm, thickness = 5.0 mm, Spacing = 1.5 mm, nine slices per volume (three axials, three sagittals, three coronals), matrix 256 × 128, (2) 3D SPGR anatomical sequence TI 500 ms, TR 5 ms, TE minimum (1.3 ms), flip angle 11°, bandwidth 31.25 MHz, FOV 26 × 26, slice thickness 1.1 mm, spacing 0.0, 128 slices per volume, one NEX images × two (acquisitions averaged off line), matrix 256 × 256 coronal oblique orientation, aligned to the long axis of the hippocampus, and (3) T2 fluid-attenuated inversion recovery (FLAIR): 2D IR axial images, TR = 10,000 ms, TE = 122 ms, TI = 2,000 ms, FOV = 24, matrix = 320 × 256, NEX = 1, slice thickness = 5 mm, 31 slices.

**Figure 1:**
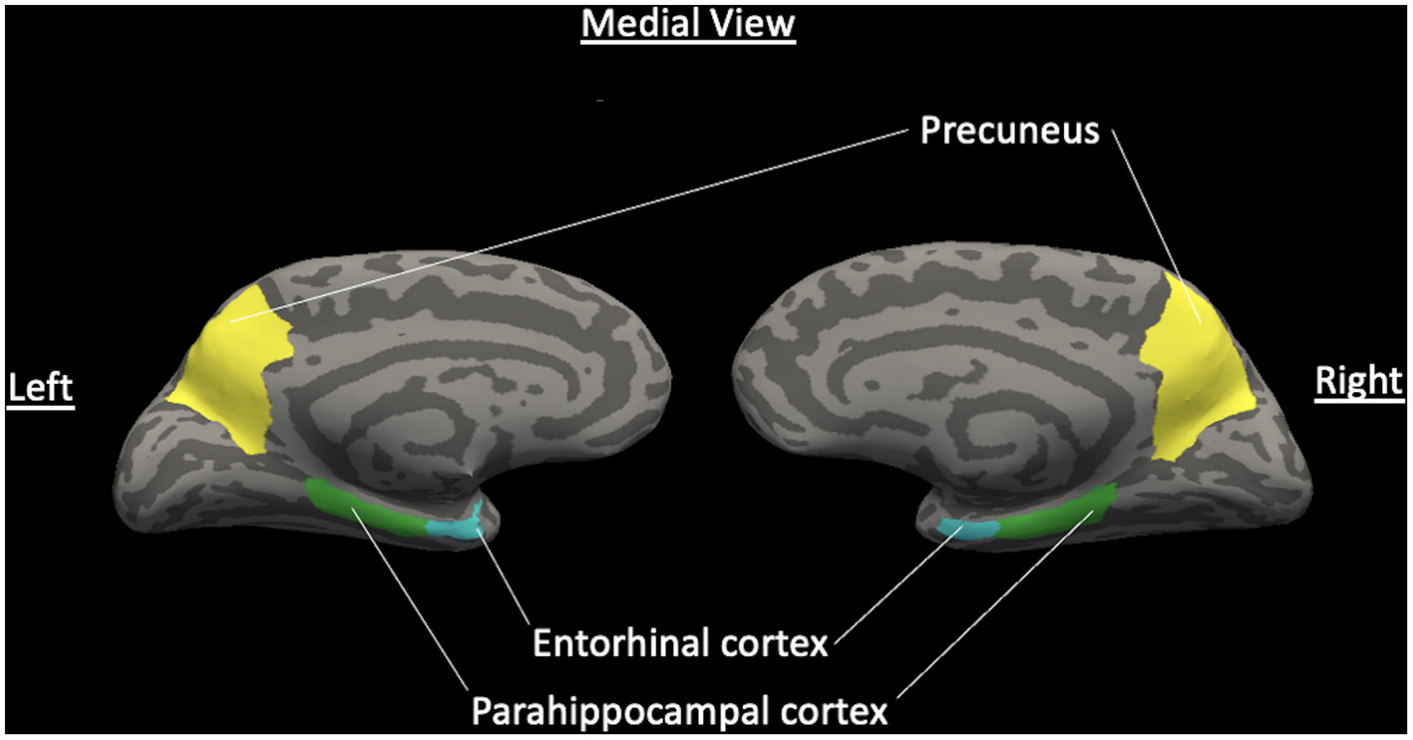
Normalized brain with ROIs of hypothesized cortical thickness differences. Medial view of a normalized brain with highlighted hypothesized regions of interest of decreased cortical thickness between VD and non-VD patients for the left and right hemispheres: entorhinal cortex, parahippocampal cortex, and precuneus gyrus. Light gray features are sulci and dark gray features are gyri (Color should be used in print for this figure).

Cortical thickness analyses were obtained using Freesurfer 5.3, a fully automated program for obtaining cortical thickness and volume. Bilateral entorhinal, parahippocampal, and precuneus cortices, as well as left and right hippocampal volume were selected as ROIs a priori using the Desikan-Killiany atlas. Figure 1 presents a normalized brain with these ROIs highlighted. Cortical thickness was computed as the distance between the white matter surface and pial surface at each location along the cortex. Hippocampal volume was also extracted using this procedure.

#### VD Classification

T2-weighted FLAIR were evaluated for the presence of DWMHs. The severity of lesions was rated by a neuroradiologist using the Fazekas modified Coffey Rating Scale for signal hyperintensities (21). DWMHs were defined as abnormalities in the frontal, parietal, temporal, or occipital lobes and scored as 0 (absent), 1 (punctate foci), 2 (beginning confluence of foci), and 3 (large confluence of foci). Patients were classified as having MRI-defined VD if they received a score of two or higher on their DWMH (6, 22). Lesion volume estimates were calculated using MRIcro for quantitative evaluation of DWMH. The full procedure for calculation using this method has been described elsewhere (8). Patients were classified quantitatively as having VD if they fell in the highest quartile of the distribution for WMH volume scores, which is consistent with previous research (8, 23, 24).

### Missing Data

The multiple imputation procedure in SPSS (25) was used to accommodate missing cortical thickness and hippocampal volume data (34.1%). This procedure replaces missing data with a set of plausible values on the basis of all variables in the data set, including demographic, clinical outcome, and neuropsychological test variables. This report is based on 20 imputed data sets (*m* = 20), which is satisfactory in obtaining excellent results unless rates of missing data are extremely high (26). The imputed datasets were analyzed using standard statistical analyses and the results of these analyses are combined using Rubin's rules (25).

### Statistical Analyses

Baseline differences in demographic and clinical variables were compared using independent *t*-tests for continuous variables and chi-square analyses for categorical variables. Cortical thickness and hippocampal volume of each hemisphere were linearly regressed on qualitative and quantitative VD status to determine differences between VD and non-VD. Cortical thickness analyses were then adjusted for site, age, sex, and mean thickness, and hippocampal volume analyses were adjusted for site, age, sex, and total intracranial volume (ICV).

#### Statistical Significance

Due to the use of multiple tests, a more stringent significance value of *p* ≤ 0.003 was used to determine statistical significance. This value was calculated in line with the Bonferroni correction method, which suggests dividing the critical alpha by the number of comparisons being made (27). As such, using the traditional significance value of *p* < 0.05, we divided this number by 16 (the number of comparisons necessitated by hemispheric ROIs for cortical thickness and hippocampal volume) to set an appropriate significance value to reduce the chances of obtaining false positives.

### Human Subjects Research

The investigation in this study was carried out in accordance with the 2013 version of the Declaration of Helsinki. The study design was review by IRB Ethics Committees at Queens College, Columbia University and the New York State Psychiatric Institute, and Harlem Hospital Center. Informed consent was obtained from all participants in the study.

## Results

### Descriptive Statistics

Forty-six participants met inclusion criteria and forty-one received structural MRI at baseline. Table 1 presents demographic and clinical data for the whole sample (*n* = 41) as well as divided into MRI-defined VD (*n* = 15) and non-VD (*n* = 26) patients (using the categorical Fazekas scale). The sample had an average age of 62.3 (SD = 9.55) years old (minimum age = 50, maximum age = 83) and was 56.1% female. There were no differences in age, sex, age of depression onset, depressive symptom severity, cumulative illness score, or cognition as measured by the MMSE. VD patients were significantly more likely to be African American, have fewer years of education, and have a lower likelihood of having a family history of a mood disorder.

**Table 1:** Baseline demographic and clinical characteristics.

**Variable**	**Total sample** **(*n =* 41)**	**MRI-defined VD** **(*n =* 15)**	**Non-VD** **(*n =* 26)**	**t/Chi square statistic**
	**M (SD) or *n* (%)**	**M (SD) or *n* (%)**	**M (SD) or *n* (%)**	
Site (%)^*^				χ^2^ = 9.90, *p* = 0.002
HHC	17 (41.5%)	11 (73.3%)	6 (23.1%)	
NYSPI	24 (58.5%)	4 (26.7%)	20 (76.9%)	
Age (years)	62.29 (9.55)	63.13 (11.06)	61.81 (8.87)	*t* = −0.42, *p* = 0.672
Women (%)	23 (56.1%)	11 (73.3%)	12 (46.2%)	χ^2^ = 2.85, *p* = 0.091
Race (%)^*^				χ^2^ = 12.69, *p* = 0.013
Caucasian	19 (46.3%)	2 (13.3%)	17 (65.4%)	
African American	18 (43.9%)	11 (73.3%)	7 (26.9%)	
Hispanic	2 (4.9%)	1 (6.7%)	1 (3.8%)	
Education^*^	14.95 (2.98)	13.47 (2.85)	15.72 (2.76)	*t* = 2.58, *p* = 0.014
Age at depression onset (years)	41.65 (21.16)	37.43 (23.45)	43.92 (19.93)	*t* = 0.91, *p* = 0.364
FH mood disorder^*^	22 (55.9%)	4 (28.6%)	20 (75%)	χ^2^ = 7.20, *p* = 0.007
HRSD	23.32 (5.64)	23.40 (7.21)	23.27 (4.66)	*t* = −0.07, *p* = 0.944
CIRS-G total score	3.91 (3.30)	4.92 (2.84)	3.33 (3.47)	*t* = −1.34, *p* = 0.180
MMSE	28.49 (1.33)	28.40 (0.99)	28.54 (1.50)	*t* = 0.36, *p* = 0.722
DWMH volume^*^	1.09 (1.79)	2.65 (2.22)	0.18 (0.24)	*t* = −4.28, *p* < 0.001

* *HHC, Harlem Hospital Center; NYSPI, New York State Psychiatric Institute; FH, family history; HRSD, Hamilton Rating Scale for Depression; CIRS-G, Cumulative Illness Rating Scale—Geriatrics; MMSE, Mini Mental Status Exam; DWMH, deep white matter hyperintensity*.

### Cortical Thickness and Hippocampal Volume Between Groups

Tables 2, 3 present the comparison of group means between VD and non-VD patients, with VD status defined qualitatively (Fazekas) and quantitatively (WMH volume). Analyses of group means showed no statistical differences in cortical thickness in the precuneal, entorhinal, and parahippocampal cortices regardless of hemisphere between VD and non-VD patients (*p* > 0.003). Similarly, differences in group means for left and right hippocampal volume did not meet statistical significance. Significance levels did not change when adjusting for site, age, sex, and mean thickness or ICV. Despite not reaching statistical significance, the difference between left entorhinal thickness in VD vs. non-VD yielded a medium effect size (defined categorically, Cohens *d* = 0.55; defined continuously, Cohens *d* = 0.41), suggesting that VD patients have decreased left entorhinal thickness compared to non-VD patients.

**Table 2:** Comparison of mean cortical thickness and hippocampal volume between MRI-defined VD and non-VD patients (Fazekas rating scale).

**ROI**	**VD** **(*n =* 15) Mean (SD)**	**Non-VD** **(*n =* 26) Mean (SD)**	***B* (SE)**	**Effect size (Cohen's *d*)**
L entorhinal	3.00 (0.29)	3.17 (0.33)	−0.19 (0.14), *p* = 0.181	0.55
R entorhinal	3.37 (0.43)	3.37 (0.53)	0.001 (0.20), *p* = 0.998	0.00
L parahippocampal	2.54 (0.26)	2.58 (0.28)	−0.02 (0.12), *p* = 0.888	0.15
R parahippocampal	2.49 (0.31)	2.53 (0.27)	−0.02 (0.12), *p* = 0.836	0.14
L precuneus	2.22 (0.16)	2.21 (0.16)	0.01 (0.06), *p* = 0.843	0.06
R precuneus	2.21 (0.12)	2.21 (0.17)	−0.01 (0.06), *p* = 0.863	0.00
L hippocampal volume	3544.09 (475.09)	3442.92 (372.03)	100.09 (133.71), *p* = 0.454	0.24
R hippocampal volume	3725.11 (483.14)	3588.29 (417.23)	135.98 (143.37), *p* = 0.343	0.30

* ROI, region of interest; L, left hemisphere; R, right hemisphere; VD, vascular depression; non-VD, non-vascular depression. Unadjusted values did not change when adjusted for age, gender, site, mean thickness, and total intracranial volume.

**Table 3:** Comparison of mean cortical thickness and hippocampal volume between quantitatively-defined VD and non-VD patients (DWMH volume).

**ROI**	**VD** **(*n =* 10) Mean (SD)**	**Non-VD** **(*n =* 31) Mean (SD)**	***B* (SE)**	**Effect size (Cohen's *d*)**
L entorhinal	3.00 (0.30)	3.13 (0.35)	−0.14 (0.15), *p* = 0.374	0.41
R entorhinal	3.33 (0.50)	3.38 (0.47)	−0.05 (0.21), *p* = 0.806	0.11
L parahippocampal	2.60 (0.27)	2.57 (0.28)	0.03 (0.12), *p* = 0.837	0.09
R parahippocampal	2.58 (0.32)	2.51 (0.28)	0.08 (0.12), *p* = 0.534	0.26
L precuneus	2.22 (0.17)	2.22 (0.16)	0.01 (0.07), *p* = 0.918	0.04
R precuneus	2.20 (0.12)	2.20 (0.15)	−0.003 (0.06), *p* = 0.963	0.02
L hippocampal volume	3520.57 (429.96)	3467.35 (409.62)	53.22 (150.75), *p* = 0.724	0.13
R hippocampal volume	3700.68 (433.14)	3617.76 (449.34)	82.93 (162.13), *p* = 0.609	0.19

*ROI, region of interest; L, left hemisphere; R, right hemisphere; VD, vascular depression; non-VD, non-vascular depression. Unadjusted values did not change when adjusted for age, gender, site, mean thickness, and total intracranial volume.

## Conclusions

To our knowledge, this is the first study directly comparing cortical thickness and hippocampal volume in depressed older adults with and without MRI-defined vascular disease. No significant differences were found between vascular and non-vascular depressed patients in cortical thickness or hippocampal volume. The lack of statistical differences in thickness of medial temporal structures and precuneus gyrus and hippocampal volume in this study raises a number of diagnostic, etiological, and developmental possibilities. Of course, larger datasets, prospective longitudinal studies, and cognitively intact controls are needed to address these questions.

One possible reason for the lack of statistical differences observed is that VD is a valid subtype of LLD, etiologically distinct from other late-life psychiatric disorders. Atrophy of the medial temporal lobe and precuneus manifest prior to onset of cognitive impairment and predict later conversion to MCI and dementia in cognitively normal individuals (12, 13, 15). Since reduced cortical thickness and hippocampal volume are hallmarks of other late life diagnostic entities like MCI and dementia, the lack of statistical differences suggest that these entities are non-overlapping and lends support to the credence that VD is a distinct diagnostic entity (28). The problem with this interpretation, however, is that both groups in this sample (VD and non-VD) likely have decreased cortical thickness relative to healthy controls, as cortical thickness has been used to differentiate depressed from non-depressed elderly (17–19). Thus, without a healthy control group, conclusions cannot be drawn as to whether both groups have reduced thickness and hippocampal volume consistent with a pre-dementia profile or appropriate values of thickness and volume for depressed elderly. Additionally, it may be more challenging to identify differences between VD and non-VD as they might both be in a pre-dementia phase where differences in thickness and volume would later become more detectable.

Another possibility is that vascular disease may not be a common causal mechanism in the depression-dementia relationship. As no statistical differences in cortical thickness or hippocampal volume were found between VD and non-VD patients, data from this study are not consistent with the idea that vascular lesions are a critical etiological factor for depression and dementia. Indeed, when LLD patients are matched for vascular risk to non-depressed older adults, LLD patients show thinner frontal lobes and lower hippocampal volumes (19), suggesting the possibility that gray matter brain changes may be more related to the presence of a mood disorder than the presence of vascular disease.

It is also conceivable that no significant differences were observed between the VD and non-VD groups because white matter changes predate gray matter deterioration. The presence of elevated regional WMHs has been observed up to 20 years before symptom onset of autosomal AD (29), whereas cortical atrophy has been observed up to 11 years prior to symptom onset in AD (12). This leaves a gap of 9 years in which VD could manifest. The temporal relationship between white matter and gray matter changes in cognitively normal adults is unclear, with some studies suggesting associations between WMHs and cortical thickness (30) and others suggesting no association (31). Interestingly, healthy controls and AD patients show inverse associations between WMHs and cortical thickness, such that higher WMH volume is associated with decreased cortical thickness, but MCI patients show a positive association between the presence of WMHs and cortical thickness, such that higher WMH volume is associated with an increase in cortical thickness values (30). This demonstrates a contradictory relationship during the transition between intact cognition and conversion to AD, which may be due to neuroinflammation stimulated by WMHs or AD pathology (30). Thus, it is plausible that VD may be a prodromal phase occurring somewhere between white matter deterioration and gray matter atrophy. The cross-sectional nature of the data in the study does not allow us to test for this possibility.

The lack of statistical differences noted also speaks to the heterogeneity of how VD has been defined. The VD hypothesis was first formulated based on patients with depression onset past the age of 60 and clinical evidence of vascular disease (score ≥ 1 on vascular scale of Cumulative Illness Rating Scale—Geriatrics) (4). Steffens & Krishnan defined VD as depression onset after age 50 with a DWMH qualitative rating ≥2 or neuropsychological impairment (executive dysfunction) (5). Alexopoulos and colleagues broadened their VD criteria into Depressive-Executive Dysfunction (DED) Syndrome, where participants were diagnosed solely on the presence of depression and executive dysfunction (32). Krishnan et al. later proposed a diagnosis of MRI-defined subcortical ischemic depression (used in the present study), where the only criteria was depression and a DWMH rating ≥2 or a subcortical gray hyperintensity (SGH) rating of 3 (22), for which both internal (6) and external (8) validity have been provided. Quantitative approach to classification using lesion volume, as used in this study, has also been advocated (23, 24). Despite the heterogeneity of this construct, supplemental analyses were run using these five definitions of VD and results were consistent between definitions, indicating no significant differences in thickness or hippocampal volume between VD and non-VD patients (see Supplementary Tables 1, 2). Similarly, significant associations were not found between MMSE score, MRI-defined VD status, DWMH lesion volume, or left entorhinal thickness (see Supplementary Data and Supplementary Table 3).

Of further consideration is that although WMHs and executive dysfunction are predictive factors in antidepressant treatment non-response, the VD hypothesis does not establish a mechanistic explanation for treatment non-response. The known mechanisms of action for Selective Serotonin Reuptake Inhibitors (SSRIs) relates to salutary modulation of hyperactive limbic structures (33) and stimulation of neurogenesis (34), and SSRIs are effective in preventing and treating post-stroke depression (35). Therefore, it is unclear why vascular damage to frontostriatal tracts would prevent SSRIs from working effectively, when they are effective in treating post-stroke depression. The level of specificity in terms of the mechanism of antidepressant non-response, the diagnostic overlap between VD, MCI, and those diagnosed with MCI and depression, the measurement of components that define the construct, and the complexity of the syndrome as it is currently understood, as well as the interaction between these components, require more precision if we are to determine the causal mechanism of VD.

This study was limited in the size of the sample. Because of this, it is important to consider effect sizes in addition to statistical significance. Notably, the difference in left entorhinal thickness values between VD and non-VD yielded a medium effect size (Cohens *d* = 0.55), suggesting that VD patients showed decreased thickness in the left entorhinal cortex compared to non-VD. This is consistent with the hypothesis that VD may be a transitional state between white matter and gray matter deterioration, as decreased entorhinal thickness has been suggested as a biomarker for AD in the prodromal phase (36), and the majority of studies investigating cortical thickness in MCI/dementia implicate the left hemisphere as opposed to bilateral thinning (12, 14, 37). This medium effect sized difference between VD and non-VD in the left entorhinal cortex supports the possibility that VD may in fact be a prodromal phase of MCI or AD.

Another limitation of this study is the young patient population, encompassing individuals over the age of 50, as opposed to more traditional older adult populations of 60+ or 65+, which may be more consistent with the MCI literature. The use of ages >50 in this study reflects the common use of age >50 in the VD literature (5, 21). Additionally, gray matter atrophy is but one measure used in addressing conversion to MCI or AD, and this study was not able to evaluate other contributing variables prevalent in this population, such as amyloid-beta and tau retentions. Additional longitudinal research is required to supplement these limitations, as the cross-sectional nature of this study does not allow for investigation of diagnostic conversion. These limitations are potentially offset by important methodological strengths, including examination of gray matter atrophy in a population where these variables have not been traditionally studied, and half of the sample consisting of African Americans, who have been shown to be at greater risk for VD than Caucasians (9). Unfortunately, the small sample size limits an investigation into the role of race in VD. Nonetheless, this is one of the first studies to date using cortical thickness and hippocampal volume to compare vascular and non-vascular depressed geriatric patient profiles.

## Data Availability Statement

The raw data supporting the conclusions of this article will be made available by the authors, without undue reservation.

## Ethics Statement

The studies involving human participants were reviewed and approved by IRB Ethics Committees at Queens College, Columbia University and the New York State Psychiatric Institute, and Harlem Hospital Center. The patients/participants provided their written informed consent to participate in this study.

## Author Contributions

DE was responsible for data analyses. DE, SS, BR, and JS are responsible for data interpretation and drafting the manuscript with input from all authors. JM and AG are responsible for the generation of cortical thickness and hippocampal volume data. JS was responsible for study conception and design. All authors contributed to the article and approved the submitted version.

## Conflict of Interest

The authors declare that the research was conducted in the absence of any commercial or financial relationships that could be construed as a potential conflict of interest.
