# Supplementary material for: Cortical Thickness and Hippocampal Volume in Vascular and Non-vascular Depressed Patients
Source: Front Psychiatry. 2021 Jul 14;12:697489. doi: 10.3389/fpsyt.2021.697489 (PMC8316761; doi:10.3389/fpsyt.2021.697489)
Supplement: Supplementary file 1 [file Data_Sheet_1.docx]

Supplementary Material

# Supplementary Data

There was no significant difference in MMSE between DWMH Fazekas ratings, *F*(3,37)=.84, *p*=.483 (see Table 3 for means). No association was found between MMSE and DWMH when WMH volume was evaluated quantitatively, *r*=.03, *p*=.850. Further, when VD was classified according to lesion volume (highest quartile lesion volume= VD), there were no significant differences in MMSE between VD (M=28.40, SD=1.17) and non-VD (M=25.82, SD=1.39), *t*(39)=.24, *p*=.813. In both the VD and non-VD group (classified according to lesion severity with the Fazekas scale), no significant associations were found between MMSE and DWMH lesion volume (VD: *r*=.21, *p*=.469; non-VD: *r*=-0.02, *p*=.922), MMSE and left entorhinal thickness (VD: *r*=.05, *p*=.872; non-VD: *r*=.09, *p*=.762), or DWMH lesion volume and left entorhinal thickness (VD: *r*=.02, *p*=.950; non-VD: *r*=.10, *p*=.691).

# Supplementary Figures and Tables

| **Table 1. Five Definitions of Vascular Depression** | |
| --- | --- |
| **Definition** | **Criteria** |
| 1. Vascular Depression (Alexopoulos et al., 1997) | Clinical evidence of vascular disease (CIRS-G vascular scale ≥ 1) |
| 2. Vascular Depression (Steffens & Krishnan, 1998) | DWMH ≥ 2 or neuropsychological impairment (executive dysfunction) |
| 3. Depression-Executive Dysfunction syndrome | Executive dysfunction |
| 4. Subcortical Ischemic Depression /MRI-Defined VD | DWMH ≥ 2 or SCH = 3 |
| 5. Quantitative Lesion Volume | Volumetric ratings of DWMH in 75^th^ percentile or higher |

*CIRS-G=Cumulative Illness Rating Scale-Geriatrics, DWMH=deep white matter hyperintensities, SCH=subcortical grey hyperintensities.

**Table 2. Comparison of mean cortical thickness and hippocampal volume across groups.**

|  | | **ROI** | **Mean (SD)** | **Mean (SD)** | ***B* (SE)** | **Effect Size (Cohen’s *d*)** |
| --- | --- | --- | --- | --- | --- | --- |
| Alexopoulos  et al., 1997  Criteria | | | **VD (n=11)** | **Non-VD (n=30)** |  |  |
|  | | L entorhinal | 3.13 (.29) | 3.09 (.36) | .04(.15), *p*=.773 | .13 |
|  | | R entorhinal | 3.36 (.36) | 3.37 (.51) | -.01(.19), *p*=.996 | .02 |
|  | | L parahippocampal | 2.60 (.28) | 2.57 (.27) | .03(.12), *p*=.219 | .09 |
|  | | R parahippocampal | 2.51 (.31) | 2.53 (.29) | -.02(.14), *p*=.906 | .05 |
|  | | L precuneus | 2.15 (.13) | 2.24 (.16) | -.09(.06), *p*=.185 | .58 |
|  | | R precuneus | 2.18 (.14) | 2.21 (.15) | -.03(.05), *p*=.573 | .23 |
|  | | L HV | 3518.40 (387.29) | 3466.38 (423.32) | 52.02(146.18), *p*=.722 | .13 |
|  | | R HV | 3705.26 (351.25) | 3613.32 (473.29) | 91.94(156.97), *p*=.558 | .22 |
| Steffens & Krishnan, 1998 Criteria |  | | **VD (n=21)** | **Non-VD (n=20)** |  |  |
|  | L entorhinal | | 3.03 (.32) | 3.17 (.35) | -.14(.14), *p*=.324 | .40 |
|  | R entorhinal | | 3.37 (.44) | 3.36 (.52) | .01(.18), *p*=.944 | .03 |
|  | L parahippocampal | | 2.54 (.28) | 2.62 (.27) | -.08(.10), *p*=.436 | .29 |
|  | R parahippocampal | | 2.50 (.32) | 2.56 (.26) | -.06(.11), *p*=.563 | .21 |
|  | L precuneus | | 2.22 (.16) | 2.21 (.15) | .01(.06), *p*=.801 | .09 |
|  | R precuneus | | 2.19 (.13) | 2.21 (.16) | -.02(.06), *p*=.728 | .14 |
|  | L HV | | 3476.76 (453.74) | 3484.09 (369.96) | -7.33(129.73), *p*=.955 | .02 |
|  | R HV | | 3615.16 (525.92) | 3661.95 (343.63) | -46.80(139.57), *p*=.737 | .11 |
| DED Criteria |  | | **DED (n=18)** | **Non-DED (n=23)** |  |  |
|  | L entorhinal | | 3.04 (.34) | 3.15 (.34) | -.11(.14), *p*=.419 | .33 |
|  | R entorhinal | | 3.37 (.45) | 3.37 (.50) | .01(.18), *p*=.969 | .01 |
|  | L parahippocampal | | 2.54 (.30) | 2.60 (.26) | -.07(.10), *p*=.521 | .24 |
|  | R parahippocampal | | 2.46 (.32) | 2.57 (.26) | -.11(.11), *p*=.294 | .38 |
|  | L precuneus | | 2.20 (.15) | 2.23 (.16) | -.03(.06), *p*=.66 | .16 |
|  | R precuneus | | 2.18 (.14) | 2.22 (.15) | -.04(.06), *p*=.547 | .25 |
|  | L HV | | 3433.32 (446.78) | 3517.13 (384.61) | -83.81(129.98), *p*=.519 | .20 |
|  | R HV | | 3534.19 (466.09) | 3719.22 (413.35) | -185.03(137.63), *p*=.179 | .42 |
| SID/ MRI-Defined VD |  | | **VD (n=15)** | **Non-VD (n=26)** |  |  |
|  | L entorhinal | | 3.00 (.29) | 3.17 (.33) | -.19(.14), *p*=.181 | .55 |
|  | R entorhinal | | 3.37 (.43) | 3.37 (.53) | .001(.20), *p*=.998 | 0.00 |
|  | L parahippocampal | | 2.54 (.26) | 2.58 (.28) | -.02(.12), *p*=.888 | .15 |
|  | R parahippocampal | | 2.49 (.31) | 2.53 (.27) | -.02(.12), *p*=.836 | .14 |
|  | L precuneus | | 2.22 (.16) | 2.21 (.16) | .01(.06), *p*=.843 | .06 |
|  | R precuneus | | 2.21 (.12) | 2.21 (.17) | -.01(.06), *p*=.863 | 0.00 |
|  | L HV | | 3544.09 (475.09) | 3442.92 (372.03) | 100.09(133.71), *p*=.454 | .24 |
|  | R HV | | 3725.11 (483.14) | 3588.29 (417.23) | 135.98(143.37), *p*=.343 | .30 |
| Highest Quartile DWMH Volume |  | | **VD (n=10)** | **Non-VD (n=31)** |  |  |
|  | L entorhinal | | 3.00 (.30) | 3.13 (.35) | -.14(.15), *p*=.374 | .41 |
|  | R entorhinal | | 3.33 (.50) | 3.38 (.47) | -.05(.21), *p*=.806 | .11 |
|  | L parahippocampal | | 2.60 (.27) | 2.57 (.28) | .03(.12), *p*=.837 | .09 |
|  | R parahippocampal | | 2.58 (.32) | 2.51 (.28) | .08(.12), *p*=.534 | .26 |
|  | L precuneus | | 2.22 (.17) | 2.22 (.16) | .01(.07), *p*=.918 | .04 |
|  | R precuneus | | 2.20 (.12) | 2.20 (.15) | -.003(.06), *p*=.963 | .02 |
|  | L HV | | 3520.57 (429.96) | 3467.35 (409.62) | 53.22(150.75), *p*=.724 | .13 |
|  | R HV | | 3700.68 (433.14) | 3617.76 (449.34) | 82.93 (162.13), *p*=.609 | .19 |

*ROI=region of interest, L=left hemisphere, R=right hemisphere, HV=hippocampal volume, DED=Depression Executive Dysfunction, SID=Subcortical Ischemic Depression, DWMH=Deep white matter hyperintensity. Unadjusted values did not change when adjusted for age, gender, site, mean thickness, and total intracranial volume.

| **Table 3. MMSE Score and DWMH Severity** | |
| --- | --- |
| DWMH Severity on Fazekas Scale | MMSE Mean (SD) |
| 0 | 29.17 (1.60) |
| 1 | 28.35 (1.46) |
| 2 | 28.20 (1.03) |
| 3 | 28.80 (.84) |

*MMSE = Mini-Mental Status Exam; DWMH = deep white matter hyperintensities.
